# Clinical and prognostic differences between methicillin-resistant and methicillin-susceptible *Staphylococcus aureus* infective endocarditis

**DOI:** 10.1186/s12879-020-4895-1

**Published:** 2020-02-21

**Authors:** Carmen Hidalgo-Tenorio, Juan Gálvez, Francisco Javier Martínez-Marcos, Antonio Plata-Ciezar, Javier De La Torre-Lima, Luis Eduardo López-Cortés, Mariam Noureddine, José M. Reguera, David Vinuesa, Maria Victoria García, Guillermo Ojeda, Rafael Luque, José Manuel Lomas, Jose Antonio Lepe, Arístides de Alarcón

**Affiliations:** 1 Department of Infectious Diseases, Hospital Universitario Virgen de las Nieves, Av. de las Fuerzas Armadas n° 2, 18014 Granada, Spain; 2 Infectious Disease Service, Hospital Universitario Virgen de la Macarena, Sevilla, Spain; 3 Infectious Disease Service, Hospital Juan Ramón Jiménez, Huelva, Spain; 4 Infectious Disease Service, Hospital Regional Universitario Carlos Haya, Málaga, Spain; 5 Internal Medicine Service, Hospital Costa del Sol, Marbella, Spain; 6 Infectious Disease Unit, Hospital Universitario San Cecilio, Granada, Spain; 7 Infectious Disease Service, Hospital Universitario Virgen de la Victoria, Málaga, Spain; 8 Infectious Disease Service, Hospital Universitario Virgen del Rocío, Sevilla, Spain; 9 Infectious Disease Service, Oxford University Hospitals NHS Foundation Trust, Oxford, UK

**Keywords:** Endocarditis, *Staphylococcus aureus*, Vancomycin, Methicillin resistance

## Abstract

**Background:**

*S. aureus* (SA) infective endocarditis (IE) has a very high mortality, attributed to the age and comorbidities of patients, inadequate or delayed antibiotic treatment, and methicillin resistance, among other causes. The main study objective was to analyze epidemiological and clinical differences between IE by methicillin-resistant versus methicillin-susceptible *SA* (MRSA vs. MSSA) and to examine prognostic factors for SA endocarditis, including methicillin resistance and vancomycin minimum inhibitory concentration (MIC) values > 1 μg/mL to MRSA.

**Methods:**

Patients with SA endocarditis were consecutively and prospectively recruited from the Andalusia endocarditis cohort between 1984 and January 2017.

**Results:**

We studied 437 patients with SA endocarditis, which was MRSA in 13.5% of cases. A greater likelihood of history of COPD (OR 3.19; 95% CI 1.41–7.23), invasive procedures, or recognized infection focus in the 3 months before IE onset (OR 2.9; 95% CI 1.14–7.65) and of diagnostic delay (OR 3.94; 95% CI 1.64–9.5) was observed in patients with MRSA versus MSSA endocarditis.

The one-year mortality rate due to SA endocarditis was 44.3% and associated with decade of endocarditis onset (1985–1999) (OR 8.391; 95% CI (2.82–24.9); 2000–2009 (OR 6.4; 95% CI 2.92–14.06); active neoplasm (OR 6.63; 95% CI 1.7–25.5) and sepsis (OR 2.28; 95% CI 1.053–4.9). Methicillin resistance was not associated with higher IE-related mortality (49.7 vs. 43.1%; *p* = 0.32).

**Conclusion:**

MRSA IE is associated with COPD, previous invasive procedure or recognized infection focus, and nosocomial or healthcare-related origin. Methicillin resistance does not appear to be a decisive prognostic factor for SA IE.

## Background

The incidence of infective endocarditis (IE) is low in industrialized countries (3–9 cases per 100,000 people-years) but has recently increased. This rise has been attributed to improvements in diagnostic methods, an increase in life expectancy, and a higher rate of instrumentalization (e.g., pacemaker and central venous catheter implantation, hemodialysis, etc.) in an increasingly aged and fragile population [1]. The most common type of IE is native valve endocarditis (largely mitral or aortic) [2]. *Staphylococcus aureus* (SA) is one of the most frequently involved bacteria [3, 4] and is associated with high morbidity and mortality rates due to its strong avidity for endothelial tissue, its capacity to produce endovascular infection, and its aggressive character [5, 6]. A European study of hospitalized patients with bacteremia by SA reported a higher risk of 30-day mortality in those infected with methicillin-resistant *S. aureus* (MRSA) versus methicillin-susceptible *S. aureus* (MSSA) [OR of 1.8(95%CI, 1.04 to 3.2)] [7]. Besides methicillin resistance, poor prognostic factors for bacteremia by SA include the presence of IE, comorbidities [8], inadequate antibiotic treatment [9], and a vancomycin minimum inhibitory concentration (MIC) > 1 μg/mL [10].

The objective of this study was to determine differences in epidemiological, clinical, and prognostic variables between MRSA versus MSSA IE and to analyze the prognostic value of vancomycin MIC > 1 μg/mL for MRSA.

## Methods

We conducted a prospective, multicenter, longitudinal study of consecutive patients with IE hospitalized in eight hospitals of the Health Service of Andalusia (Spain) between 1984 and 2017. During this period, these hospitals prospectively enrolled a total of 2076 patients with IE from whom informed verbal consent to study participation was obtained. Ethics committee approved this procedure.

Among these patients, we prospectively enrolled in the present cohort the 437 patients with *S. aureus* endocarditis who met eligibility criteria. Information was prospectively gathered by attending physicians on epidemiological, clinical, analytical, and prognostic (mortality, relapse) data and on medical and surgical treatments. Heart surgery was available at five of the eight hospitals, to which candidates for surgery from the other three hospitals were transferred. Patients were followed up for 12 months after IE, monitoring clinical, analytical, and microbiological results. The EuroSCORE, logistic EUROSCORE, and modified Duke Criteria were calculated for all patients and included as study variables when they became incorporated into clinical practice, with the agreement of the endocarditis study group, as well as data on new antibiotics. There were no changes during the study period in the other study parameters, i.e., clinical, epidemiological, microbiological, analytical findings, duration of antibiotic therapy, performance of surgery, adequate antibiotic treatment (according to contemporary recommendations), or mortality data.

All information was treated in accordance with national legislation on personal data protection (Organic Law 15/1999, 13 December, of Personal Data Protection), and the study was approved by the ethics committee of the coordinating center (Hospital Universitario Virgen del Rocio, Seville).

### Inclusion criteria

Inclusion criteria were: age ≥ 18 years with “definite” or “possible” *S. aureus* IE according to modified Duke Criteria [11], which were retrospectively applied to patients enrolled before their publication.

### Definitions

*Previous valve disease* included any rheumatic, congenital, degenerative/myxoid, or degenerative/calcified valve disease in the patient’s clinical records.

*History of invasive procedure or previous infection focus* included previous dental manipulation (extraction or other invasive dental or maxillofacial technique) or invasive urinary or vascular procedure (e.g., central/peripheral catheterization) and/or the presence of a focus of infection (urinary, cutaneous, vascular, etc.) in the 3 months before the IE episode.

*Central nervous system (CNS) symptoms* included encephalopathy, meningitis, brain abscess, hemorrhagic or ischemic embolism, and transient stroke.

*Acute kidney failure during hospitalization* was defined by creatinine > 1.5 mL or 25% increase versus baseline.

*The age-adjusted Charlson comorbidity index* was used to estimate the 10-year life expectancy of our patients as a function of age and comorbidities [12], determined at admission to hospital for the endocarditis episode.

*Surgical risk* was estimated at admission in all patients using EuroSCORE I or II (*European System for Cardiac Operative Risk Evaluation),* which predicts early mortality after cardiac valve surgery [13, 14]. We used both scores in order compare their capacity to predict the prognosis. In cases of IE between 1998 and 2017, this scale was calculated prospectively by the attending physician. In cases of IE before 1998, the EuroSCORE was calculated retrospectively from data in the clinical records.

*IE relapse* was defined by an episode of *S. aureus* endocarditis within 12 months of a first episode that had met cure criteria.

*IE reinfection* was defined by a second IE due to a microorganism other than SA during the follow-up year.

*Mortality rates* considered deaths for any cause during hospitalization or the first 30 days post-discharge *(hospital mortality)* as well as the *IE-related mortality* [e.g., heart failure due to valve dysfunction] and the *non-IE-related mortality* [e.g., cancer] at 1 year post-discharge**.**

*Severe sepsis* was defined by ≥2 criteria of systemic inflammatory response syndrome with organ dysfunction; and *Septic shock* by sepsis with refractory hypotension and end-organ perfusion dysfunction despite adequate fluid resuscitation [15].

*Early prosthetic IE* was defined by onset during the first year post-surgery and *Late prosthetic IE* by onset after more than 1 year post-surgery [16].

*Nosocomial IE* was defined by symptom onset more than 48 h after hospital admission [17].

*Healthcare-related IE* was defined by symptom onset more after medical manipulation in the 3 months preceding the diagnosis (intravenous treatments, wound healing, hemodialysis, and stays at care home or assisted centers) [17].

The indication for surgery was initially assessed by the attending physician, based on universally accepted criteria at the time [18–20], and was confirmed by consensus of a multidisciplinary team that included attending physicians and heart surgeons, who also considered the quality of life, comorbidities, surgical prognosis, and life expectancy of patients.

Indications for surgery were divided into five groups: a) Surgery not indicated; b) Surgery indicated and performed without delay; c) Surgery indicated and performed with delay > 72 h in grade IV left ventricular failure (LVF) or delay > 1 week in progressive LVF; d) Surgery indicated but not performed for any cause (e.g., technical impossibility, neurological complication, death before surgery, patient refusal, etc.); and e) Surgery indicated but not proposed by the attending physician due to the condition of patients (e.g., comorbidity with low life expectancy, critical status, etc.).

*Postponed surgery* was defined by its performance after 1 month of hospitalization.

*Methicillin resistance* was defined by an inhibition halo for oxacillin of ≤10 mm or oxacillin MIC ≥4 mg/L. The E-test with oxacillin strip was used in some centers [21] and automated microdilution systems in others [22].

*Adequacy of antibiotic treatment* was defined by its accordance with antibiogram results and its recommendation for IE in clinical practice guidelines.

*Diagnostic delay* was defined by an interval of ≥7 days between symptom onset and first hospital consultation.

### Statistical analysis

In a descriptive analysis, central tendency and dispersion measures (mean, standard deviation, median, percentiles) were calculated for quantitative variables and absolute frequencies with 95% confidence interval (CI) for qualitative variables. In bivariate analyses, prognostic, clinical, epidemiological, and therapeutic variables were compared between patients with MRSA IE versus MSSA IE, and mortality rates for SA endocarditis were compared with prognostic factors. The Student’s t-test for independent samples was used for quantitative variables with a normal distribution and the Mann-Whitney U test for those with non-normal distribution. Qualitative variables were analyzed using Pearson’s or Fisher’s chi-square test, as appropriate. The normality of variable distribution was checked with the Kolmogorov-Smirnov test. Two multivariate logistic regression models were developed according to Freeman’s formula [*n* = 10*(k + 1)] [23], one for differences between MRSA IE versus MSSA IE and the other for factors related to mortality in patients due to SA IE. The models included variables found to be statistically significant in bivariate analyses or considered clinically relevant. A stepwise procedure was used, considering an entry probability of 0.05 and exit probability of 0.10. Goodness of fit was evaluated with the Hosmer-Lemeshow test. The regression model for differences between MRSA versus MSSA IE included the following variables: history of myocardiopathy, congenital heart disease, hemodialysis, chronic obstructive pulmonary disease (COPD); intravenous drug addiction; Charlson index; early prosthetic IE; perivalvular involvement diagnosed by echocardiography; previous invasive procedure or focus of infection; place of IE acquisition; interval between symptom onset and hospital admission; cutaneous manifestations (Osler’s nodes, Janeway lesions); Duke vascular or embolic phenomena; and adequate treatment administration. The regression model for risk factors associated with mortality due to IE included: hospital where IE was treated, decade of IE onset, previous valve disease, early prosthetic IE, IE on pacemaker or defibrillator lead, IE on mitral valve, onset of IE as severe sepsis/septic shock, CNS involvement, kidney failure, heart failure, infectious osteoarticular involvement (arthritis/osteomyelitis), surgical risk (EuroSCORE, logistic EuroSCORE), heart surgery indicated without delay and performed during hospitalization, and surgery indicated but not performed. IBM SPSS Statistics 20.0 software was used for data analyses. The level of significance was 0.05 for all tests.

## Results

### Differences in epidemiological, clinical, and prognostic variables between MRSA versus MSSA IE

We included 378 patients with MSSA IE and 59 with MRSA IE from 1984 through January 2017 (15.8% 1984–1999; 35.7% 2000–2009, and 48.5% 2010–2017).

According to bivariate analyses, the main epidemiological and clinical differences between IE by MRSA versus MSSA were age (62.5 vs. 58 years, *p* = 0.048); nosocomial acquisition (71.2 vs. 41.8%; *p* = 0.001); history of COPD (30.5 vs. 12.2%; *p* = 0.0001), elevated Charlson index (3 [1–4] vs. 2 [0–3]; *p* = 0.006), history of previous invasive procedure or focus (79.7 vs. 57.1%; *p* = 0.001), and more frequent diagnostic delay (57.7 vs. 39.5%, *p* = 0.05). In comparison to patients with MRSA IE, higher percentages of those with MSSA IE had congenital heart disease (7.1 vs. 0%; *p* = 0.037), were in a hemodialysis program (7.6 vs. 0%; *p* = 0.02), and had infectious perivalvular involvement during IE (28.3 vs. 15.3%; *p* = 0.043). The remaining results are listed in Tables 1 and 2.

**Table 1:** Epidemiology and history of MRSA vs. MSSA endocarditis (bivariate analysis)

	MSSA IE *N* = 378	MRSA IE *N* = 59	p*
Mean age (yrs), (± DS)	58.05. (±17.8)	62.3. (±15)	**0.048**
Females, n (%)	133 (35.2)	21 (35.6)	0.95
Males	245 (64.8)	38 (64.4)	
Native IE, n (%)	295 (78)	44 (74.6)	0.55
Early prosthetic IE, n (%)	20 (5.3)	7 (11.9)	0.07
Late prosthetic IE, n (%)	39 (10.3)	4 (6.7)	0.39
IE on device (AICD, PMK), n (%)	34 (8.9)	5 (8.4)	0.89
Valve involved, n (%)			
- Mitral	209 (55.3)	31 (52.5)	0.8
- Aortic	129 (34.1)	16 (27.1)	0.34
- Mitro-aortic	17 (4.2)	4	0.5
- Mitral, aortic, and tricuspid	3 (0.8)	0	1
- Mitral and tricuspid or Aortic and tricuspid	16 (4.2)	1 (1.7)	0.7
- Tricuspid	46 (12.1)	7 (11.8)	0.99
- Pulmonary	4 (1.05)	1(1.6)	0.51
- IE on interventricular communication, n (%)	4 (1.05)	0	1
Acquisition setting, n (%)			
Community	221(58.5)	17(28.8)	
Nosocomial	128(33.9)	37(62.7)	**0.0001**
Healthcare-related	29(7.7)	5(8.5)	
Decade of endocarditis onset, n (%)			
1985–1999	61 (16.1)	7(11.8)	0.4
2000–2009	136 (35.9)	20 (33.9)	0.756
2010–2017	180 (47.6)	32 (54.2)	0.344
History of: n (%)			
- previous IE	18 (4.8)	7(11.9)	0.64
- valve disease on native valve	171 (45.2)	24(40.7)	0.42
- Rheumatic	51 (13.5)	7(11.9)	0.68
- Myxoid degeneration and/or mitral prolapse	26 (6.9)	3 (5.1)	0.78
- Degenerative/calcified	58 (15.3)	10 (16.9)	0.8
- Congenital valve disease	27 (7.1)	0	**0.037**
- Heart disease	98 (25.9)	21(35.6)	0.81
- Cardiomyopathy	35(9.3)	12(20.4)	**0.01**
- COPD	46(12.2)	18(30.5)	**0.0001**
- Diabetes Mellitus	91 (24.1)	18 (30.5)	0.22
- Peptic ulcer	5 (1.3)	3(5.1)	0.12
- Arterial hypertension	123 (32.5)	26(44.1)	0.09
- Peripheral vascular disease	21(5.6)	6(10.2)	0.24
- Stroke	21(5.6)	5(8.5)	0.37
- Dementia	6(1.6)	2(3.4)	0.3
- Active neoplasm	30 (7.9)	6(10.2)	0.61
- Colonic polyposis	8 (2.1)	4 (6.8)	0.13
- Transplant (*)	5 (1.3)	0	1
- Chronic liver disease, n (%)	49 (12.9)	6 (10.2)	0.54
- Child-Pugh A	29 (59.2)	1(16.7)	
- Child-Pugh B	3(6.1)	1(16.7)	0.7
- Child-Pugh C	3(6.1)	1(16.7)	
- Hemodialysis	29 (7.7)	0	**0.02**
- Kidney failure	80 (21.2)	11(18.6)	0.6
-IVDA	38(10.1)	2(3.4)	0.097
Charlson index, median (IQR)	2(0–3)	3(1–4)	**0.006**
Previous invasive procedure or infection focus, n (%)	216 (57.1)	47(79.7)	0.001
Vascular	148 (68.5)	27(57.4)	0.63
Urinary (catheter)	7(3.2)	4(8.5)	0.08
Abdominal	3(1.4)	2(4.3)	0.19
Dental	5(2.3)	0	1
Locomotor	6(2.8)	3(6.4)	0.17

*COPD* Chronic Obstructive Pulmonary Disease; *IVDA* intravenous drug addiction; Kidney failure: creatinine clearance increase of > 1.5 mL/min or 25% versus baseline. Transplant (*): 3 kidney, 1 heart, 1 hematopoietic progenitor

**Table 2:** Comparison of clinical and echocardiographic findings between MRSA versus MSSA endocarditis (bivariate analysis)

	MSSA IE *N* = 378	MRSA IE *N* = 59	p*
Findings < 1 week before hospitalization, n (%)	212/355 (59.7)	22/52 (42.3)	0.051
Clinical n (%)			
**-** Fever	365(96.6)	55(93.2)	0.251
- Dyspnea	174(46)	31(52.5)	0.361
- Constitutional syndrome	81(21.4)	11(18.6)	0.689
- Murmur	199(52.6)	30(50.8)	0.822
- Hepatomegaly	66(17.4)	8(13.6)	0.420
- Splenomegaly	48(12.7)	9(15.3)	0.615
- CNS	156(41.3)	18(30.5)	0.116
Encephalopathy	76 (20.1)	9 (15.3)	0.4
Meningitis	20 (5.3)	1 (1.69)	0.3
Abscess	6 (1.6)	0	1
Embolic non-hemorrhagic stroke	62 (16.4)	7 (11.9)	0.43
Embolic hemorrhagic stroke	11 (2.9)	2 (3.39)	0.68
Hemorrhagic stroke with no previous Embolism	22 (5.8)	2 (3.39)	0.75
- Renal embolism	6(1.6)	0	1
- Spleen embolism	22(5.8)	5(8.5)	0.387
- Large vessel embolism	30(7.9)	5(8.5)	0.799
- Pulmonary embolism	27(7.1)	8(13.6)	0.119
- Roth’s spots	13 (3.4)	0	0.228
- Conjunctival hemorrhage	15(3.9)	0	0.230
- Endophthalmitis	0	1 (1.7)	0.127
- Cutaneous manifestation	108 (28.6)	11 (18.6)	0.093
- Petechiae	80 (21.2)	7 (11.9)	0.115
- Janeway lesions	53(14)	3(5.1)	**0.054**
- Osler’s nodes	61(16.1)	4(6.8)	**0.057**
- Splinter hemorrhage	43(11.4)	5(8.5)	0.496
- Duke vascular or embolic phenomena	120(31.7)	12(20.4)	0.072
- Duke immunological phenomena	29 (7.7)	1 (1.7)	0.099
- Osteoarticular involvement	52(13.8)	8(13.6)	0.98
- Acute kidney failure during hospitalization	159(42)	22(37.3)	0.469
- Heart failure	171 (45.2)	25(42.4)	0.643
Grade III-IV	90 (23.8)	18 (30.5)	0.3
- Severe sepsis/Septic shock	97(25.7)	14(23.7)	0.751
- Acute pulmonary edema	93(24.6)	17(28.8)	0.446
Echocardiographic findings, n (%) **			
- Diagnostic data	336 (88.9)	49(83.1)	0.34
- Vegetation	249(65.9)	43 (72.9)	0.615
- Perivalvular lesion	107(28.3)	9(15.3)	**0.043**
- Pseudoaneurysm	14(3.7)	1(1.7)	0.7
- Fistula in valvular system	8(2.1)	2(3.4)	0.63
- Valvular system rupture or perforation	63(16.7)	6(10.2)	0.226
- Prosthesis dehiscence or dysfunction	23(6.1)	5(8.5)	0.39
- Pericardial effusion	14(3.7)	2(4.4)	1
IE classification, n(%)			
Possible	25 (6.6)	7(11.9)	
Definite	352(93.1)	52(88.1)	0.18

(**) Transthoracic Echocardiogram was obtained in 94.9% of patients with MRSA IE and transesophageal echocardiogram in 54.2%. Transthoracic echocardiogram was obtained in 96.3% of patients with MSSA IE and transesophageal echocardiogram in 56.3%

With respect to outcomes, bivariate analysis showed a significantly longer hospitalization in patients with MRSA versus MSSA endocarditis (37 vs. 30 days; *p* = 0.019) but no significant difference in mortality from IE at 1 year (49.1 vs. 43.7%, *p* = 0.33) or in the percentage of patients undergoing surgery (25.4 vs. 30.9%; *p* = 0.13). Among the patients with MRSA IE, there was a larger percentage for whom surgery was indicated but not performed (18.6 vs. 10.1%; *p* = 0.05) and a higher relapse rate (10 vs. 3%; *p* = 0.05) (Table 3).

**Table 3:** Prognosis, adequacy of antibiotic therapy, and surgical outcomes in MRSA versus MSSA IE

	MSSA IE *N* = 378	MRSA IE *N* = 59	p*
Mortality at 1 year, n (%)	165 (43.7)	29 (49.1)	0.32
Mortality at 1 year not related to IE, n (%)	13 (4.2)	0	0.316
Mortality attributable to SA IE in hospital or during the first 30 days post-discharge, n (%)	152 (40.2)	29 (49.1)	0.222
Mortality attributable to IE in the following periods, n (%)			
1984–1999	35 (23)	2 (6.99)	0.122
2000–2009	64 (42.1)	12 (41.4)	0.215
2010–2017	52 (34.2)	15 (51.7)	0.062
Reinfection, n (%)	6 (1.5)	0	0.316
IE relapse, n (%)	8 (3.1)	4(6.8)	0.053
Adequacy of antibiotic therapy, n (%)	302/332 (91)	40/46 (87)	0.085
Days of antibiotic therapy, median (IQR)	32 (19–44.5)	41 (20–62)	0.219
Hospital stay (days), median (IQR)	30 (16–47)	37 (21–58)	**0.019**
Surgery not indicated n (%)	128 (33.9)	18 (30.5)	0.189
Surgical treatment on admission, n (%)	127 (32)	15 (25.4)	0.13
Postponed surgery after discharge, n (%)	8 (2.1)	0	0.61
Surgery indicated and conducted without delay, n (%)	117 (30.9)	12 (20.3)	0.11
Surgery indicated and conducted with delay: > 72 h in left ventricular failure grade IV, n (%)	9 (2.4)	3 (5.1)	0.21
Surgery indicated and not conducted in hospital, n (%)	88 (23.3)	18 (30.5)	0.23
Surgery indicated but not conducted, n (%)	38 (10.1)	11 (18.6)	0.052
Surgery indicated but not conducted due to poor clinical status, n (%)	50 (13.2)	7 (11.86)	0.7

p* < 0.05 = significant

The risk factors associated with MRSA in the multivariate analysis were: history of COPD (OR 3.19; 95% CI 1.41–7.23), previous invasive procedure or recognized focus of infection in the three-month period before IE onset (OR 2.9; 95% CI 1.14–7.65), and a delay of ≥7 days between symptom onset and hospital admission (OR 3.94; 95% CI 1.64–9.5) (Table 4).

**Table 4:** Results of the multivariate analysis of MRSA vs. MSSA endocarditis

	OR	95% CI
COPD	**3.19**	**1.4–7.23**
Early prosthetic IE	2.13	0.69–3.98
Nosocomial or healthcare-related IE	1.64	0.69–3.99
Cardiomyopathy	2.22	0.84–5.91
Congenital disease	0	0
Arterial hypertension	1.16	0.54–2.49
Hemodialysis	0	0
Charlson’s index	0.92	0.776–1.095
Invasive procedure and/or focus of infection	**2.95**	**1.14–7.65**
IVDA	1.18	0.21–6.66
Osler’s node	0.73	0.163–3.24
Janeway lesion	0.737	0.137–3.96
Duke vascular or embolic phenomena	0.19	0.716–5.493
Delay in hospital care	**3.94**	**1.64–9.468**
Echocardiography with perivalvular lesion	0.395	0.15–1.03
Adequate antibiotic therapy	0.532	0.214–1.321

*COPD* Chronic Obstructive Pulmonary Disease; *IVDA* intravenous drug addiction

### Prognostic value for IE of vancomycin MIC> 1 μg/mL for MRSA

MIC values were determined by E-test in 37 (62.7%) of MRSA cases and by microdilution in 22 (37.3%). Vancomycin MICs for MRSA were available in 74.6% (44/59) of cases and were > 1 μg/mL in 38.6% (17/44). There were 4 relapses, observing 3 (75%) in strains with MIC > 1 μg/mL and 1 (25%) with MIC ≤1 (*p* = 0.7). The Mortality attributable to MRSA IE in hospital or during the first 30 days post-discharge was 49.1% (29/59) of which Vancomycin AUC:CMI was available in 18 patients. The hospital mortality rate was 61.1% (11/18) in strains with MIC ≤1 μg/mL versus 38.8% (7/18) with MIC> 1 μg/mL, also a non-significant difference (*p* = 0.847).

### Risk factors associated with mortality from SA

During the hospital stay or within 30 days post-discharge, 182 (44.8%) patients died from SA endocarditis-specific mortality. The mortality rate was 56.9% (37/65) in 1985–1999; 53.8% (78/145) in 2000–2009; and 34.2% (67/196) in 2010–2017. During the one-year follow-up, 13 (2.9%) died from a cause other than IE, 6 (1.4%) died from a new IE episode due to a resistant strain, and 16 (3.7%) were lost to the follow-up. Data were available on the treatment of 378 patients (86.5%), and 342 (90%) of these received adequate initial antibiotic treatment according to the antibiogram and clinical practice guidelines.

The mortality from SA in our cohort was associated in bivariate analyses with: the decade of endocarditis onset (1985–1999: mortality rate of 20.3% (37/182) vs. survival rate of 12.5% (28/224), *p* = 0.032; 2000–2010: 42.8% (78/182) vs. 36.8% (67/224), *p* = 0.007; 2010–2017: 36.8% (67/182) vs 57.6% (129/224); *p* = 0.0001); older age (61.5 vs. 56.4 years; *p* = 0.004); active neoplasm (11.2 vs. 5.8%; *p* = 0.05); early prosthetic IE (9.4 vs. 4%; *p* = 0.028); mitral valve involvement (64.6 vs. 49.5%; *p* = 0.03); sepsis/septic shock (46.7 vs. 27.8%; *p =* 0.0001); kidney failure during IE episode (52.2 vs. 33.9%; *p* = 0.0001); de novo heart failure (60.2 vs. 33.8%; *p* = 0.0001); CNS involvement (encephalopathy 37.6 vs. 22.6%; *p* = 0.006; embolic stroke 29.6 vs. 18.7%; *p* = 0.033; meningitis 8.9% vs 5.8%; *p* = 0.035); high surgical risk (median EuroSCORE of 13 vs. 9; *p =* 0.0001; median logistic EuroSCORE of 30.76 vs. 15.4; *p* = 0.0001**;** and indication but non-performance of surgery (16.5 vs. 4.9%; *p =* 0.0001). MRSA itself did not emerge as a risk factor for mortality in our cohort (29% vs. 11.8%, *p* = 0.22) Protective factors were: IE on pacemaker lead or automatic implantable cardioverter-defibrillator (AICD) (2.8 vs. 12.9%; *p =* 0.0001); osteoarticular spread of the infection (9.6 vs. 17.3%; *p* = 0.026); heart surgery conducted when indicated without delay (23.1 vs. 36.2%; *p* = 0.004). According to the multiple logistic regression analysis, poor prognostic factors for SA endocarditis were: decade of endocarditis onset in 1985–1999 (OR 8.391; 95% CI (2.82–24.9) or 2000–2009 (OR 6.4; 95% CI 2.92–14.06); active neoplasm (OR 6.63; 95% CI 1.7–25.5); and sepsis/shock (OR 2.28; 95% CI 1.053–4.9) (Table 5).

**Table 5:** Risk factors associated with mortality in SA endocarditis. Results of bivariate and multivariate analyses

	Death *N* = 182	Survivors *N* = 224	p*	OR (95% CI); p*
Age (yrs), media (± DS)	61.5 (±16.83)	56.4 (±17.75)	**0.004**	1.02(0.997–1.042); 0.09
Females, n (%)	66 (36.3)	76 (33.9)	0.62	
Native IE, n (%)	146(80.2)	168(75)	0.21	
Early prosthetic IE, n (%)	17(9.4)	9 (4)	**0.028**	1.307(0.395–4.328); 0.661
Late prosthetic IE, n (%)	22 (12.2)	20(8.9)	0.29	
IE in devices (AICD, PMK), n (%)	5 (2.8)	29(12.9)	**0.0001**	0.34(0.06–2.12); 0.252
Affected valve, n (%)				
Mitral	115 (64.6)	110 (49.5)	**0.03**	1.456 (0.742–2.86); 0.274
Aortic	61 (34.1)	73 (32.9)	0.83	
Tricuspid	21 (11.7)	30 (13.5)	0.59	
Pulmonary	4 (2.2)	0	**0.025**	
Mitral and aortic	9 (4.9)	12 (5.3)	0.91	
Mitral, aortic, and tricuspid	2 (1.1)	1(0.04)	0.59	
Mitral and tricuspid	10 (5.5)	6 (2.6)	0.19	
Community acquisition setting, n (%)	93 (51.1)	129 (57.6)	0.181	
Decade of endocarditis onset, n (%)				
1985–1999	37 (20.3)	28 (12.5)	**0.0032**	8.391(2.82–24.95); 0.0001
2000–2009	78 (42.9)	67(29.9)	**0.007**	6.41 (2.921–14.06); 0.0001
2010–2017	67 (36.8)	129 (57.6)	**0.0001**	
Hospital where IE was treated, n (%)				
HUVR, (*n* = 156)	75 (41.2)	81 (36.2)		
HUVM, (*n* = 47)	18 (9.9)	29 (12.9)		
HURM, (*n* = 63)	28 (15.4)	35 (15.6)		
HUVV, (*n* = 54)	24 (13.2)	30 (11.4)	0.189	
HCS, (n = 18)	8 (4.4)	10 (4.5)		
HJRJ, (*n* = 23)	15 (8.2)	8 (3.6)		
HUSC, (*n* = 11)	5 (2.7)	6 (2.7)		
HUVN, (*n* = 34)	9 (4.9)	25 (11.2)		
History of, n (%):				
- Previous IE	11 (4.9)	12(36.6)	0.473	
- Previous valve disease	90 (51.7)	92(42.2)	**0.060**	0.846(0.437–1.64);0.621
Rheumatic	33 (19.4)	22(10.2)	0.011	
Myxoid	8 (4.7)	20(9.3)	0.085	
Degenerative/calcified	36 (21.2)	27(12.6)	0.023	
Congenital	6 (3.5)	16(7.4)	0.101	
- Heart disease	110 (60.4)	123(54.9)	0.288	
- Acute myocardial infarction previous to IE	8 (4.4)	12(5.4)	0.674	
- Auricular fibrillation	20(11.1)	17(7.6)	0.223	
- Cardiomyopathy	17 (9.4)	26(11.6)	0.484	
- COPD	31(17.2)	27(12.1)	0.141	
- Diabetes mellitus	49(27.2)	52(23.2)	0.355	
- Hypertension	56(30.8)	84(37.7)	0.146	
- Peripheral vascular disease	9(5)	16(7.1)	0.336	
- Stroke	14(7.8)	12(5.4)	0.149	
- Dementia	5(2.8)	2(0.9)	0.25	
- Active neoplasm	20(11.2)	13 (5.8)	**0.051**	6.627(1.72–25.53); 0.006
- Kidney failure	38 (21)	47(21)	0.998	
- Hemodialysis	12(6.6)	16 (7)	0.86	
- Liver disease	22 (12.2)	29 (12.9)	0.811	
- Child-Pugh A	10 ()	18 ()		
- Child-Pugh B	5()	4 ()	0.553	
- Child-Pugh C	2 (1.1)	2 (0.9)		
- HIV infection	4(2.2)	7(3.2)	0.76	
- IVDA	15(8.3)	23(10.3)	0.508	
-Transplant (*)	0	5(2.2)	0.068	
Charlson’s index, median (IQR)	4 (2–5)	2 (0.9–4)	0.084	
History of invasive procedure or previous focus, n (%)	115 (63.2)	133 (59.6)	0.46	
Going to hospital during first 7 days of symptom onset, n (%)	110 (63.6)	105(51.5)	**0.018**	
Adequate antibiotic treatment, n (%)	139(76.4)	183 (81.6	0.362	
Severe sepsis/septic shock, n (%)	85(46.7)	62(27.8)	**0.0001**	2.286(1.053–4.96); 0.037
Manifestations in CNS, n (%)	93 (51.1)	73 (32.6)	**0.0001**	0.878(0.433–1.778); 0.717
- Encephalopathy	47 (37.6)	35 (22.6)	0.006	
- Meningitis	11 (8.9)	9 (5.8)	0.035	
- Brain abscess	0	6 (3.9)	0.027	
- Embolic stroke	37 (29.6)	29 (18.7)	0.033	
- Hemorrhagic stroke with no previous embolism	14 (11.3)	8 (5.2)	0.059	
Renal embolism, n (%)	5 (2.8)	1 (0.4)	0.092	
Large vessel embolism, n (%)	13 (7.3)	18 (8.1)	0.775	
Spleen embolism, n (%)	11(6.2)	16 (7.2)	0.693	
Kidney failure during IE (**), n (%)	94 (52.2)	6 (33.9)	**0.0001**	1.279 (0.67–2.44); 0.455
Heart failure, n (%)	109 (60.2)	75 (33.8)	**0.0001**	1.65(0.773–3.523); 0.196 0.846(0.312-
Osteoarticular dissemination, n (%)	17 (9.6)	38 (17.3)	**0.026**	2.295); 0.743
MRSA, n (%)	30 (16.5)	27 (12.1)	0.201	
Surgery performed, n (%)	50(27.5)	82(36.6)	**0.051**	1.778 (0.299–10.592); 0.527
Surgery indicated and conducted without delay, n (%)	42(23.1)	81 (36.2)	**0.004**	0.242(0.041–1.426); 0.117
Surgery indicated and conducted with delay > 48 h in left ventricular failure, n (%)	9 (4.9)	2(0.89)	**0.015**	
Surgery indicated and not conducted, n (%)	30(16.5)	11(4.9)	**0.0001**	2.866(0.936–7.707); 0.066
Early surgery < 2 weeks, n(%)	24 (48)	47 (57.3)	0.68	
EuroSCORE, median (IQR)	13 (9.5–16)	9(7–12)	**0.0001**	1.038(0.862–1.25); 0.692
Logistic EuroSCORE, median (IQR)	30.76(14.7–58.7)	15.4(8.05–28.1)	**0.0001**	1.033(0.997–1.069);0.07
Relapse, n (%)	5 (2.7)	9 (4)	0.480	
Reinfection, n (%)	0	6 (2.6)	1	

P*: < 0.05 significant; OR, 95% CI

**Kidney failure** **(Creatinine > 1.5 mL or 25% increase versus baseline). Transplantation* (3 kidney, 1 heart, 1 bone marrow). **Postponed:** conducted ≥1 month of hospitalization

## Discussion

In this study of patients with *S. aureus* endocarditis, 13.5% were MRSA, all elderly men with multiple diseases, elevated Charlson index, and high surgical risk. Around half of the patients with MRSA had prior valve disease, which was rheumatic or degenerative in one-third of cases and of nosocomial origin or healthcare-related in the remainder. The IE predominantly involved left-sided native valves (predominantly mitral valves) and less frequently cardiac devices, although such cases have become increasingly frequent. The mortality rate was very high, with around half of the patients dying from IE in hospital or within 30 days of their discharge. These data are similar to previous reports associating endocarditis with high morbidity and mortality rates and linking its acquisition to health care in up to 30% of cases [24]. These trends have been attributed to the increasing incidence of aortic valve disease in elderly populations, with a greater use of valve prostheses and intracardiac devices [25]. The main risk factors for in-hospital mortality in patients with IE were recently reported to be *SA* etiology, high Charlson index score, and EuroSCORE II ≥ 9 [26]. In our patients with MRSA endocarditis, surgery was considered appropriate in 41 patients but was only carried out in 15 (36.5%), being ruled out in 7 patients due to their poor clinical status. The percentage of patients who receive surgery when indicated ranged between 15 and 45% in a recent review of *S. aureus* and endocarditis; this review reported contradictory data on the benefits of early surgery, observing that surgery was sometimes delayed for weeks or months beyond the end of antibiotic IE treatment to correct the valve damage responsible for heart failure [27].

The main differences between MRSA and MSSA endocarditis in our study population was the greater frequency of the former in patients with COPD and its association with a longer interval between symptom onset and hospital admission (≥ 7 days in around 60% of cases). One reason may be the higher MRSA colonization rate in patients with COPD due to their repeated contact with the health care system [28]. The exacerbation of COPD, mainly during the winter, is one of the principal causes of hospitalization and is often responsible for iatrogenesis, adverse effects, and functional decline [29]. This may be an important reason for the delay in hospital care.

Previous invasive procedures and/or infectious foci were also more frequent in the patients with MRSA versus MSSA IE, with 70% of the former being of nosocomial origin. There were no significant differences between MRSA and MSSA IE in surgical intervention or mortality rates or in the receipt of adequate antibiotic therapy. Many authors have associated mortality due to SA with methicillin resistance. A recent meta-analysis of 62 studies of bacteremia (13 of IE alone) found a higher mortality risk for MRSA versus MSSA, with an OR of 2.65 (95% CI, 1.46–4.80) [6]. It should be noted that most published studies on the role of methicillin resistance in the prognosis of *S. aureus* bacteremia do not include cases of endocarditis. It should also be acknowledged that *t*he prognosis of IE is influenced by numerous factors; therefore, the added prognostic value of data on methicillin resistance and vancomycin MIC may be limited*.* The association of methicillin resistance with higher treatment failure rate in our cohort did not reach statistical significance, possibly due to the low relapse rate after IE treatment with prolonged antimicrobial therapy and frequent removal of the infection focus (heart surgery).

The importance of vancomycin susceptibility in methicillin-resistant and even methicillin-susceptible strains is controversial. After initial studies described worse outcomes for methicillin-resistant strains with high vancomycin MIC values [9, 30], various meta-analyses on the relevance of MIC in SA infections have associated values ≥2 μg/mL with higher mortality (OR 1.72; 95% CI: 1.34–2.21) and values ≥1.5 μg/mL with treatment failure (OR 2.69; 95% CI:1.60–4.51) [10]. These associations have been observed not only in MRSA but also in *Staphylococcus* coagulase-negative IE with vancomycin MIC ≥2 μg/mL [31] and even in IE [32] and bacteremia due to MSSA with MIC ≥1.5 μg/mL, which was associated with a higher risk of complicated bacteremia [33, 34]. In contrast, a longitudinal, prospective, multicenter study of MRSA endocarditis found no association of vancomycin MIC ≥1.5 μg/mL with higher mortality, although it was related to a greater persistence of bacteremia and a higher frequency of sepsis/septic shock, peripheral embolism, and arthritis/osteomyelitis [35]. Likewise, a study on beta-lactam-treated left-sided MSSA endocarditis found no relationship between vancomycin MIC and mortality or microorganism virulence [36].

The mortality rate of *S. aureus* endocarditis was very high in our cohort, despite the receipt of antibiotic treatment that accorded with antibiogram results and was recommended in available clinical practice guidelines by 90% of the patients. Active neoplasm, sepsis/shock, and decade of endocarditis onset (1985–1999 or 2000–2009 vs. 2010–2017) emerged as poor prognostic factors, but early surgery (within first 2 weeks) did not appear to influence the prognosis. A recent multicenter, longitudinal, observational study of SA IE (*n* = 213 cases) reported a mortality rate of 37% and identified a high Charlson index, congestive heart failure, CNS involvement, and sepsis/septic shock as risk factors [35].

With regard to the possible beneficial effect of early surgery in SA endocarditis, a meta-analysis reported a lower mortality rate when the surgery was conducted within the first 2 weeks rather than later in cases of native IE (OR = 0.46, 95% CI [0.31, 0.69]; *p* = 0.001) but not in cases of prosthetic IE (OR = 0.83, 95% CI [0.65, 1.06]; *p* = 0.413) [37]. Another study found no reduction in one-year mortality in patients with *S. aureus* IE on prosthetic valve when the surgery was performed during the first 60 days of hospitalization rather than later (risk ratio, 0.67 [95% CI: 0.39–1.15]; *p* = 0.15). The authors therefore recommended that surgery be considered on a case-by-case basis, regardless of whether SA is involved [38].

One study limitation is that our analysis considered data gathered over three decades rather than shorter time periods in order to obtain adequate statistical power. Strengths include the large patient sample and its prospective longitudinal multi-center design, involving specialist hospitals that formed a specific study group for this purpose. The results provide a reliable understanding of the current state of endocarditis in our region and may possibly be extrapolated to other regions of our country.

## Conclusion

*S. aureus* endocarditis has a very high mortality rate in our setting. MRSA IE is associated with COPD, previous invasive procedure or recognized infection focus, and nosocomial or healthcare-related origin. Although methicillin resistance does not appear to have a decisive influence on the mortality risk, it may increase the therapeutic failure rate among patients receiving recommended treatments.

## Data Availability

The dataset used and analyzed during the current study are not publicly available because this could result in identification of patients who gave interviews on the condition of anonymity. Data are available from the corresponding author on reasonable request for researches.

## Abbreviations

AICD: Automatic implantable cardioverter-defibrillator
CNS: Central nervous system
COPD: Chronic obstructive pulmonary disease
IE: Infective endocarditis
LVF: Left ventricular failure
MIC: Minimum inhibitory concentration
MRSA: Methicillin-resistant SA
MSSA: Methicillin -susceptible SA
SA: *S. aureus*
